# Decision coaching for healthy women with *BRCA1/2* pathogenic variants and open family planning: Impact on decisional conflict and decision status: Subgroup analyses from a randomized controlled trial

**DOI:** 10.1371/journal.pone.0354884

**Published:** 2026-08-17

**Authors:** Claudia Stracke, Sibylle Kautz-Freimuth, Arim Shukri, Anna Isselhard, Birte Berger-Höger, Anke Steckelberg, Frank Vitinius, Nicola Dikow, Marion Kiechle, Cornelia Meisel, Achim Wöckel, Marion Tina von Mackelenbergh, Rita Schmutzler, Kerstin Rhiem, Stephanie Stock

**Affiliations:** 1 Faculty of Medicine, University of Cologne and Institute for Health Economics and Clinical Epidemiology, University Hospital Cologne, Cologne, Germany; 2 Institute for Health and Nursing Science, Medical Faculty of Martin Luther University Halle-Wittenberg, University Medicine Halle, Halle (Saale), Germany; 3 Institute for Public Health and Nursing Research, University of Bremen, Bremen, Germany; 4 Faculty of Medicine, University of Cologne and Department of Psychosomatics and Psychotherapy, University Hospital Cologne, Cologne, Germany; 5 Department of Psychosomatic Medicine, Robert Bosch Hospital, Stuttgart, Germany; 6 Institute for Human Genetics, University Hospital Heidelberg, Heidelberg, Germany; 7 Department of Obstetrics and Gynecology, TUM University Hospital, Technical University of Munich, Munich, Germany; 8 Department of Gynecology and Obstetrics, Medical Faculty and University Hospital Carl Gustav Carus, Technische Universität Dresden, Dresden, Germany; 9 National Center for Tumor Diseases/University Cancer Center (NCT/UCC): German Cancer Research Center (DKFZ), Heidelberg, Germany; 10 Faculty of Medicine and University Hospital Carl Gustav Carus, Technische Universität Dresden, Helmholtz-Zentrum Dresden-Rossendorf (HZDR), Dresden, Germany; 11 Department of Obstetrics and Gynecology, University Hospital Würzburg, Würzburg, Germany; 12 Department of Obstetrics and Gynecology, University Hospital Schleswig–Holstein, Kiel, Germany; 13 Faculty of Medicine, University of Cologne and Center for Familial Breast and Ovarian Cancer, University Hospital Cologne, Cologne, Germany; Odense University Hospital, DENMARK

## Abstract

**Background:**

Women with *BRCA1*/*2* pathogenic variants (PVs) without a personal cancer history and with open family planning are in a very complex decision-making situation regarding their preventive and reproductive options. This exploratory study aims to assess the impact of a decision coaching (DC) program on decisional conflict and decision-making of these women regarding their preventive decision.

**Methods:**

*BRCA1/2* PV carriers without a personal cancer history were recruited and randomly assigned to intervention or control group (IG, CG). The IG participated in a DC program consisting of in-person decision coaching and an evidence-based, structured decision aid about the preventive options (intensified breast surveillance, risk-reducing bilateral mastectomy, and risk-reducing bilateral salpingo-oophorectomy); the CG received usual care. Data was collected at baseline, 12 weeks and 6 months after study inclusion. Decision-related outcomes regarding the participants’ preventive decision were measured with the Decisional Conflict Scale (DCS) and the Status of Decision-Making Scale. Subgroup analysis was performed to assess differences between women with open versus completed family planning using descriptive statistics and multiple linear regression models.

**Results:**

Women with open family planning experienced higher baseline DCS scores regarding their preventive decision-making than women with completed family planning (mean DCS total score 39.5 vs. 34.4; mean difference −5.12, 95%CI [−9.328;-0.977], *p = 0.018*). In the total group, decisional conflict decreased considerably more in the IG than in the CG. Among women in the IG, those with open family planning showed a greater reduction in DCS scores than women with completed family planning. In initially undecided women with open family planning, 12 weeks after study inclusion significantly more women in the IG reached a preventive decision than in the CG (61.8% vs. 23.3%, *p < 0.001*). Significantly more women with open family planning in the IG opted for the intensified breast surveillance program compared to the CG.

**Conclusions:**

A DC program developed for unaffected *BRCA1/2* PV carriers may contribute to reducing decisional conflict and increasing decision-making, especially in women with open family planning.

## 1. Background

Women with a pathogenic variant (PV) in the *BRCA1* or *BRCA2* gene are confronted with an increased lifetime risk for breast cancer (BC) and ovarian cancer (OC) of up to approximately 70% for BC and 17% (*BRCA2*) to 44% (*BRCA1*) for OC [1].

Preventive options for *BRCA1/2* PV carriers with no personal cancer history (unaffected) consist of intensified breast surveillance (IBS), risk-reducing bilateral mastectomy (RRBM), and risk-reducing bilateral salpingo-oophorectomy (RRBSO). The IBS program of the centers of the German Consortium for Hereditary Breast and Ovarian Cancer (GC-HBOC) includes biannual physical examinations and breast sonogram starting at age 25, annual MRIs from age 25, and biennial mammograms beginning at age 40 [2]. The program cannot reduce the risk of developing BC but is able to detect BC at an early stage in over 80% of the cases [3]. RRBM reduces the incidence of BC and there is first evidence for a reduced overall mortality for *BRCA1* PV carriers [4,5]. RRBSO reduces the incidence of OC as well as OC-specific and overall mortality [6–8].

However, these risk management strategies are also associated with negative long-term consequences: participation in the IBS program may be accompanied by stress, worry and ongoing anxiety and uncertainty [9]; RRBM leads to loss of the ability to breast-feed and may affect physical sensation and emotional well-being; RRBSO leads to loss of fertility and may cause surgical menopause [10].

Given these different options with the associated risks and benefits, deciding for or against a risk management strategy can be a very challenging and difficult situation for unaffected women who receive a positive genetic test result for a *BRCA1/2* PV. Studies on women’s psychological situation show that distress, anxiety and cancer worry are significantly increased in women after genetic test disclosure [11]. Beyond that, unaffected women with a *BRCA1/2* PV, who have open family planning, are in a particularly difficult situation. They do not only have to make preventive decisions, but also reproductive decisions on whether, when and how to become pregnant [12–14]. In complex decision-making situations like these, decisional conflict, defined as a state of uncertainty about a course of action, may arise and lead to decision delay or failure of decision implementation [15,16]. According to O’Connor et al. [17], people with high decisional conflicts have a poorer knowledge about the options and are more likely to express decisional regret. Decision coaching (DC) programs offer personal and non-directive support and aim to support people in their decision-making process and to activate people to participate in making health decisions [18,19]. The Ottawa Decision Support Framework (ODSF) is a theoretical framework that guides the development, implementation and evaluation of DC interventions [20].

In a recent study, Stock et al. [21] evaluated a DC program tailored for unaffected women with *BRCA1/2* PVs according to the guidelines of the ODSF, with the focus on the roles that women wish and take during their decision-making process. It has been shown that the DC program enabled women to take a more active role in decision-making [21]. Furthermore, they were more satisfied with their role taken, made their decision earlier and had a greater knowledge [21]. It is also of clinical relevance to investigate to what extent the DC program helps women to deal with their decisional conflict. Therefore, the effect of the program will be further analyzed with the focus on decisional conflict. To our knowledge, the impact of a DC program on decisional conflict in women with a *BRCA1/2* PV, particularly those with open family planning, has not yet been investigated. However, there are already studies in other medical fields that examine the influence of DC on decision-making conflicts. In a recent Cochrane systematic review by Jull et al. [19] regarding DC for people facing healthcare decisions, the authors analyzed the effects of DC plus evidence‐based information versus UC and versus evidence-based information only. For DC plus evidence-based information versus UC, the meta-analysis showed reduced scores in the DCS informed subscale but with a very low certainty of evidence. For the DCS subscales values clarity and support only one study of the meta-analysis reported reductions for the DC intervention group. Wang et al. [22] assessed the effect of a decision support intervention, consisting of counseling, DA and DC, on decisional conflict of patients with elevated serum prostate-specific antigen in a RCT. The decision support intervention significantly reduced patients’ decisional conflict (DCS total score). A study by Li et al. [23] examined the effectiveness of a nurse-led decision counseling intervention, consisting of health education, information and values clarification, aiming to improve hepatocellular carcinoma screening among patients with hepatitis B. The authors found a higher decrease of patients’ decisional conflict (DCS total score) in the IG compared to the CG receiving UC. To the best of our knowledge, there is no study focusing on the effect of DC programs on decisional conflict of unaffected *BRCA1/2* PV carriers.

The present study aims to evaluate the impact of the DC program on decisional conflict of unaffected women with *BRCA1/2* PVs and to explore possible differences that can be observed between women with open family planning and those who have completed their family planning. The exploratory hypothesis was that particularly women with open family planning could benefit from participating in the DC program in terms of decision-making and decisional conflict.

## 2. Methods

### 2.1. Study design

This exploratory subgroup analysis is based on data from the randomized controlled EDCP-BRCA trial carried out at six GC-HBOC centers [21]. The EDCP-BRCA trial investigated the effectiveness of a DC program consisting of a nurse-led in-person DC [24] plus an evidence-based decision aid (DA) [25,26], both specifically developed for healthy women with *BRCA1/2* PVs, versus usual care (UC). Primary endpoint of the EDCP-BRCA trail was the congruence between the desired and actual role taken in the decision-making process. Study registration was on 30/10/2019 under the DRKS ID: DRKS00015527.

A study protocol including the RCT’s methodology is published elsewhere [27]. In short: Female *BRCA1/2* PV carriers were asked to complete a baseline questionnaire (T1). Those returning the questionnaire were included in the study and randomized to the intervention group (IG) and the control group (CG). The IG received UC and participated in the DC program, while the CG received UC only. Further questionnaires were to be completed 12 weeks (T2) and 6 months (T3) after study inclusion. Before study start, all doctors participating in counseling completed a communication training based on the KoMPASS concept [28].

### 2.2. Ethics approval and consent to participate

Ethical approval was obtained from the University Hospital of Cologne Research Ethics committee (reference number 19–1110). Written informed consent was obtained from all participants for being included in the study.

### 2.3. Study population

Inclusion criteria were women aged 25–60 years, diagnosed with a definite *BRCA1/2* PV, not affected by BC/OC, sufficient German language skills, and written informed consent to participate in the study. The recruitment period was from 18/11/2019 to 27/10/2021. In total, 389 participants were enrolled in the original RCT, whereby 198 women were randomized to the IG and 191 to the CG.

### 2.4. Variables

#### 2.4.1. Socio-demographic and medical variables.

Socio-demographic and medical variables were assessed at baseline. Family status (single vs. in a relationship), having children (yes vs. no), employment status (employed vs. unemployed) and educational level (academic vs. non-academic) were assessed on a dichotomized scale. The category ‘non-academic’ includes women with the following educational qualifications: no school diploma, lower secondary school diploma, or intermediate secondary school diploma (including technical high school diploma). The category ‘academic’ refers to women who hold a higher education entrance qualification or a tertiary degree, such as a high school diploma or a college/university degree (including universities of applied sciences). Age was assessed on a metric scale. The PV was assessed, and women were categorized into the group *BRCA1* PV and the group *BRCA2* PV.

#### 2.4.2. Family planning.

This analysis focuses on two subgroups of women with different family planning statuses. Women’s personal position towards family planning was assessed at baseline (T1) with the question ‘Is your family planning complete?’. Women answering ‘Yes’ were categorized into the subgroup ‘completed family planning’, women who stated ‘No’ were categorized into the subgroup ‘open family planning’. The data for this subgroup analysis are based on the original RCT; however, the ‘open family planning’ and ‘closed family planning’ subgroups are examined for the first time in the present analysis.

#### 2.4.3. Outcome variables: decision-related variables.

The outcome variables of this analysis were women’s mean decisional conflict and women’s decisional status at baseline (T1), 12 weeks after study inclusion (T2) and 6 months after study inclusion (T3).

*Decisional conflict:* The German version of the decisional conflict scale (DCS) was used to assess women’s decisional conflict [15, 29]. It measures the level of decisional conflict that people experience while making health care decisions. The DCS includes five subscales (informed, values clarity, support, uncertainty, effective decision), that together form the total scale. The DCS consists of 16 items, each using a five-point Likert scale (from 0 = strongly agree to 4 = strongly disagree). The item scores are summed and linear-transformed to range from 0 (no decisional conflict) to 100 (extremely high decisional conflict). DCS total scores lower 25 are associated with implementing decisions, scores exceeding 37.5 are associated with decision delay or feeling unsure about implementing decisions [15]. Therefore, DCS scores were categorized in scores <25, 25–37.5, and >37.5 and interpreted as indicating mild, moderate, and high decisional conflict, respectively [30].

*Decision status:* The stage of decision-making scale (SDM-S) was used to assess women’s decisional status [31,32]. The version used consists of one item with four response options [29]. Women who answered ‚I have already made a choice‘ were categorized in the group ‘decided’. Women who stated ‘I have not yet thought about the options’, ‘I am considering the options’, and ‘I am close to choosing one option’ were categorized in the group ‘undecided’.

### 2.5. Statistical analysis

Descriptive statistics were calculated for socio-demographic and medical characteristics as well as decision-related outcome variables. Continuous variables were described by means and standard deviations, whereas categorical variables were summarized with absolute numbers and percentages. Cross-sectional differences between IG and CG for continuous variables were analyzed using independent-samples t-tests. To account for potential deviations from normality, bootstrap confidence intervals and p-values were additionally calculated. Cross-sectional differences between IG and CG in categorical variables were compared with chi-square tests.

Multiple linear regression models were used to analyse differences in DCS scores at different time points. One model was constructed for the total sample with the DCS total score at T1 as the dependent variable, in order to assess group differences at baseline.

In addition, two separate models were built for the DCS total score at T2 – one for the IG and one for the CG – to examine group differences within subgroups of women with open family planning and those with completed family planning. This exploratory approach allowed us to examine whether associations between family planning status and decisional conflict differed between IG and CG.

To adjust for baseline differences between women with open family planning and those with completed planning, each regression model included the following baseline characteristics as independent variables: age, family status, number of children, employment status, and education level. These variables were selected to account for potential confounding due to demographic imbalances between the subgroups.

Assumptions for multiple multivariable regression were tested prior to analysis. Model fit was evaluated using unadjusted R² values. For each model, the non-standardized beta coefficients (B), standard errors (SE), t-statistics (t), and p-values are reported. To assess internal consistency, Cronbach’s alpha was calculated for the DCS total scale and all subscales (informed, values clarity, support, uncertainty, effective decision).

In the main analysis of the EDCP-BRCA trial [21], missing data were handled using multiple imputation, and the results were confirmed in a sensitivity analysis using the non-imputed dataset. Since the present study represents an exploratory secondary subgroup analysis based on the same dataset, no additional imputation procedure was performed. Given the exploratory character of the analyses, no formal adjustment for multiple testing was applied. Exact p-values are reported, and 95% confidence intervals were estimated using bootstrapping procedures to allow robust and transparent interpretation of the findings. For all statistical tests, p-values < .05 were considered significant. All statistical analyses were performed using IBM™ SPSS™ Statistics for Windows, Version 29.0 (IBM Corp, Armonk, NY, USA).

## 3. Results

### 3.1. Study population

A total of 389 women participated in the study and were randomized to the IG (n = 198) and the CG (n = 191). Table 1 provides an overview of participants’ baseline characteristics. Since the participants were randomized to the two groups, women of the IG and CG shared similar characteristics. Of the 389 randomized participants, 358 completed T2 questionnaires and 348 completed T3 questionnaires, corresponding to missing rates of 7.9% and 10.5%, respectively.

**Table 1:** Baseline characteristics of the study population.

	Total (n = 389)	IG (n = 198, 50.9%)	CG (n = 191, 49.1%)	Difference IG-CG [95% CI]	p-value
**Age in years, mean [SD]**	35.3 [8.6]	34.9 [8.0]	35.8 [9.2]	0.984 [-0.675; 2.859]	0.261
PV, n (%)*	382 (100.0)	194 (100.0)	188 (100.0)		
*BRCA1* PV, n (%)	190 (49.7)	97 (50.0)	93 (49.5)	0.5 [-0.090;0.111]	0.924
*BRCA2* PV, n (%)	192 (50.3)	97 (50.0)	95 (50.5)		
**Family status, n (%)**	389 (100.0)	198 (100.0)	191 (100.0)		
Single, n (%)	174 (44.7)	94 (47.5)	80 (41.9)	5.6 [-0.039;0.160]	0.267
In relationship, n (%)	215 (55.3)	104 (52.5)	111 (58.1)		
**Children, n (%)**	388 (100.0)	198 (100.0)	190 (100.0)		
Yes, n (%)	194 (50.0)	93 (47.0)	101 (53.2)	6.2 [-0.156;0.045]	0.231
No, n (%)	194 (50.0)	105 (53.0)	89 (46.8)		
**Employment status, n (%)**	389 (100.0)	198 (100.0)	191 (100.0)		
Employed, n (%)	311 (79.9)	166 (83.8)	145 (75.9)	7.9 [-0.168;0.009]	0.056
Unemployed, n (%)	78 (20.1)	32 (16.2)	46 (24.1)		
**Educational level, n (%)**	387 (100.0)	196 (100.0)	191 (100.0)		
Academic, n (%)	179 (46.3)	98 (50.0)	81 (42.4)	7.6 [-0.185;0.026]	0.136
Non-academic, n (%)	208 (53.7)	98 (50.0)	110 (57.6)		
**Family planning, n (%)**	348 (100.0)	176 (100.0)	172 (100.0)		
Completed, n (%)	160 (46.0)	78 (44.3)	82 (47.7)	3.4 [-0.139;0.073]	0.524
Open, n (%)	188 (54.0)	98 (55.7)	90 (52.3)		

SD: standard deviation, PV: pathogenic variant, *PV was not documented for 7 women, IG: intervention group, CG: control group, missing values result from missing information in the questionnaires. Statistical significance: two-sided independent-samples t-test with bootstrapping.

Cronbach’s alpha was 0.955 for the DCS total scale (16 items), 0.816 for the informed subscale (3 items), 0.892 for the values clarity subscale (3 items), 0.852 for the support subscale (3 items), 0.942 for the effective decision subscale (4 items). For the uncertainty subscale (3 items), it was 0.616, which is acceptable [33].

Additionally, baseline characteristics of women with open family planning were compared with those of women with completed family planning, and several differences were found (complete data is listed in additional file A). In the subgroup with open family planning, more women were single, academic, of younger age and childless compared to women with completed family planning.

### 3.2. Decisional conflict

#### 3.2.1. Decisional conflict of the total group.

**Fig 1:**
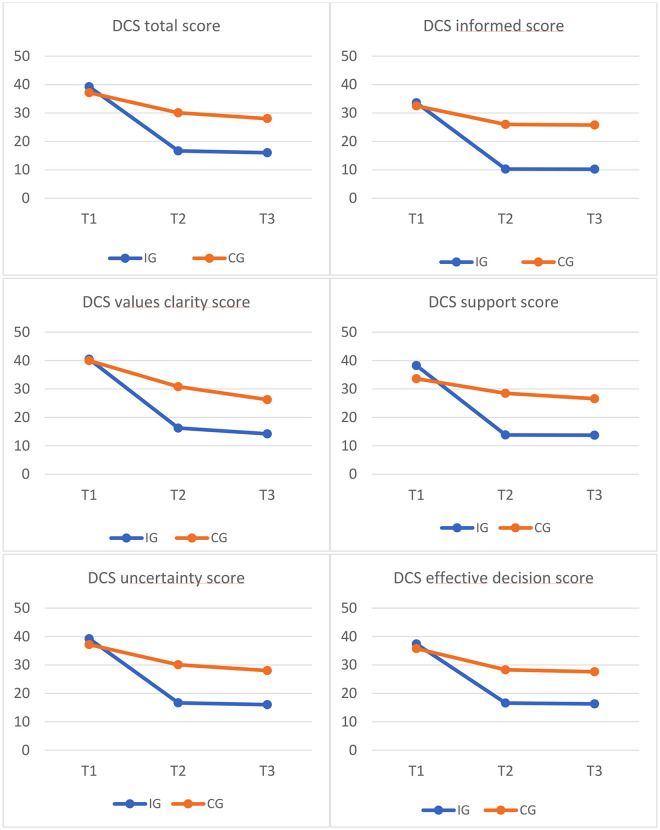
Mean DCS scores of the total group (n = 389) comparing IG and CG at baseline and after 12 weeks and after 6 months. T1: baseline, T2: 12 weeks after study inclusion, T3: 6 months after study inclusion. DCS: Decisional Conflict Scale, IG: Intervention Group, CG: Control Group. Corresponding data including all p-values in the additional file B.

Fig 1 shows the results of the mean DCS total scores and all subscores at baseline (T1), 12 weeks after study inclusion (T2) and 6 months after study inclusion (T3) for the IG and the CG of the total group.

At T1, all mean DCS scores were comparable between IG and CG.

At T2, all mean DCS scores had decreased in both IG and CG. In the IG, all mean DCS scores were significantly lower compared to the CG.

At T3, mean DCS total scores and all subscores remained almost stable in both IG and CG, and only minor further decreases were observed. All DCS scales showed significantly lower mean scores in the IG compared to CG. Complete data is listed in additional file B.

#### 3.2.2. Decisional conflict of women with open and completed family planning.

In the total group, decisional conflict of women with open family planning (n = 188) was compared to decisional conflict of women with completed family planning (n = 160) at baseline (T1) (Table 2).

**Table 2:** Decisional conflict among women in the OFP and CFP subgroups at T1.

DCS score	Total		OFP		CFP		Difference CFP-OFP [95% CI]	p-value
	n	mean [SD]	n	mean [SD]	n	mean [SD]		
Total	348	37.1 [19.5]	188	39.5 [19.2]	160	34.4 [19.7]	−5.116 [-9.328;-0.977]	**0.018**
Informed	348	32.2 [20.2]	188	35.8 [19.9]	160	28.0 [19.9]	−7,727 [-11.967;-3,311]	**<0.001**
Values clarity	348	38.4 [23.9]	188	41.4 [23.2]	160	34.9 [24.3]	−6,547 [-11.763;-1.132]	**0.015**
Support	348	35.1 [20.1]	188	35.9 [20.3]	160	34.2 [19.9]	−1.634 [-6.094;2.597]	0.462
Uncertainty	348	45.1 [24.9]	188	47.3 [24.2]	160	42.5 [25.6]	−4.764 [-10.018;0.257]	0.072
Effective decision	348	35.4 [21.2]	188	37.7 [20.9]	160	32.7 [21.3]	−4.962 [−9.432;-0.324]	**0.030**

DCS: Decisional Conflict Scale, SD: standard deviation, OFP: open family planning, CFP: completed family planning. Statistical significance: Statistical significance: two-sided independent-samples t-test with bootstrapping. Significant p-values are shown in bold.

At T1, women with open family planning had significantly higher mean scores on the DCS total scale as well as the subscales informed, values clarity and effective decision compared to women, who had completed their family planning. On the DCS total scale and the subscales values clarity, uncertainty and effective decision, the values in the subgroup with open family planning were > 37.5, which indicates a high level of conflict. Women with completed family planning had a high conflict (DCS > 37.5) in the DCS subscale uncertainty only.

Since the subgroups of women with open family planning and completed family planning differed in baseline characteristics, we used multiple linear regression models to assess differences in mean DCS scores while adjusting for potential confounders (Table 3). Visual inspection of residual plots indicated no major deviations from linearity or homoscedasticity. Residuals showed no major deviations from normality, and variance inflation factors indicated no relevant multicollinearity.

**Table 3:** Linear regression models*: DCS total score at T1.

Independent variables	R²	B	SE	t	p-value
*Univariate model*	*0.017*				
OFP^1^		5.117	2.090	2.448	**0.015**
*Multivariable model*	*0.040*				
OFP^1^		2.685	3.298	0.814	0.416
Children (yes vs. no)		1.443	2.859	0.505	0.614
Family status (single vs. in a relationship)		0.556	2.492	0.223	0.824
Employment status (unemployed vs. employed)		4.091	2.760	1.483	0.139
Educational level (non-academic vs. academic)		4.448	2.149	2.070	**0.039**
Age		−0.104	0.171	−0.606	0.545

*Dependent variable = DCS total score at T1. OFP = Open family planning ^1^Reference: completed family planning. R²: coefficient of determination, Beta: non-standardized beta coefficient, SE: standard error, t: t-statistic. Significant p-values are shown in bold.

In the univariable model, the DCS total score was significantly higher for women with open family planning (b = 5.117, SE 2.090, p = 0.015). However, in the multiple regression model adjusting for baseline characteristics (children, employment status, family status, level of education), the trend remained but the beta coefficient was not significant (b = 2.685, SE = 3.298, p = 0.416) (Table 3). Interestingly, there was a significantly positive association between educational level and DCS total score, indicating that women with an academic status had significantly higher baseline decisional conflict compared to those with a non-academic status (b = 4.448, SE = 2.149, p = 0.039).

To analyze the effect of the DC program on decisional conflict of women with open family planning and with completed family planning, IG and CG were compared within both subgroups at the time point T2. DCS scores at T3 were not included in this subgroup analysis comparing women with open family planning and completed family planning, because (i) the analysis of the mean DCS scores of the total group had shown that the main decrease in mean DCS scores and the crucial difference between IG and CG occurred at T2 and (ii) mean DCS scores at T3 remained in the same range as at T2 (see Fig 1 and additional file B).

Table 4 shows the comparison of IG and CG within the subgroup of women with open family planning.

**Table 4:** Decisional conflict of women with open family planning in the IG and CG.

DCS score		Total		IG		CG		Difference CG-IG [95% CI]	p-value
		n	mean [SD]	n	mean [SD]	n	mean [SD]		
T1	Total	188	39.5 [19.2]	98	39.4 [18.3]	90	39.5 [20.1]	0.133 [-5.392;5.658]	0.961
	Informed	188	35.8 [19.9]	98	36.5 [19.9]	90	34.9 [19.9]	−1.468 [-7.068;4.579]	0.633
	Values clarity	188	41.4 [23.2]	98	39.9 [22.3]	90	42.9 [24.0]	3.014 [-3.698;9.829]	0.393
	Support	188	35.9 [20.3]	98	37.3 [20.4]	90	34.3 [20.2]	−3.036 [-8.850;2.937]	0.285
	Uncertainty	188	47.3 [24.2]	98	46.7 [22.8]	90	47.9 [25.8]	1.238 [-5.792;8.252]	0.727
	Effective decision	188	37.7 [20.9]	98	37.3 [20.5]	90	38.1 [21.5]	0.722 [-5.279;6.624]	0.805
T2	Total	188	24.0 [18.1]	98	15.1 [11.5]	90	32.9 [19.1]	17.673 [12.926;22.671]	**<0.001**
	Informed	188	19.8 [18.7]	98	10.7 [12.3]	90	28.9 [19.5]	18.193 [13.691;23.083]	**<0.001**
	Values clarity	188	24.5 [21.9]	98	13.4 [14.3]	90	35.3 [22.9]	21.875 [16.376;27.679]	**<0.001**
	Support	188	20.6 [19.3]	98	12.0 [13.9]	90	29.2 [20.1]	17.191 [12.167;22.468]	**<0.001**
	Uncertainty	188	32.2 [22.9]	98	23.9 [17.5]	90	40.2 [24.7]	16.345 [10.076;23.044]	**<0.001**
	Effective decision	188	23.6 [19.2]	98	15.7 [14.1]	90	31.2 [20.5]	15.488 [10.063;21.089]	**<0.001**

DCS: Decisional Conflict Scale, SD: standard deviation. Statistical significance: Statistical significance: two-sided independent-samples t-test with bootstrapping. Significant p-values are shown in bold.

At T1, there were no significant differences between IG and CG in any of the mean DCS scores.

At T2, DCS scores were significantly lower in the total scale and all subscales in the IG compared to the CG. All scores in the IG had decreased to values < 25, indicating mild conflict. In the CG, all scores were > 25, indicating moderate conflict. In the DCS subscale uncertainty (mean 40.2 [24.7]) the score was even > 37.5, indicating still high conflict.

Table 5 shows the comparison of IG and CG within the subgroup of women with completed family planning.

**Table 5:** Decisional conflict of women with completed family planning in the IG and CG.

DCS score		Total		IG		CG		Difference CG-IG [95% CI]	p-value
		n	mean [SD]	n	mean [SD]	n	mean [SD]		
T1	Total	160	34.4 [19.7]	78	36.9 [18.6]	82	31.9 [20.4]	−5.001 [-11.102;0.832]	0.097
	Informed	160	28.0 [19.9]	78	27.8 [19.1]	82	28.3 [20.7]	0.474 [-5.298;6.176]	0.937
	Values clarity	160	34.9 [24.3]	78	38.7 [23.8]	82	31.2 [24.3]	−7.540 [-15.353;-0.166]	**0.049**
	Support	160	34.2 [19.9]	78	37.9 [17.6]	82	30.7 [21.5]	−7.226 [-13.151;-1.735]	**0.017**
	Uncertainty	160	42.5 [25.6]	78	45.8 [25.8]	82	39.4 [25.2]	−6.422 [-14.306;1.406]	0.119
	Effective decision	160	32.7 [21.3]	78	35.0 [20.5]	82	30.5 [21.9]	−4.487 [-10.789;2.158]	0.171
T2	Total score	160	22.7 [16.2]	78	18.7 [12.4]	82	26.7 [18.3]	7.999 [2.961;13.167]	**0.02**
	Informed	160	16.7 [17.9]	78	10.2 [12.4]	82	22.9 [19.8]	12.743 [7.429;18.641]	**<0.001**
	Values clarity	160	22.6 [19.8]	78	20.2 [16.4]	82	25.3 [22.1]	5.118 [-0.999;11.819]	0.112
	Support	160	21.9 [19.6]	78	15.6 [15.6]	82	27.4 [21.2]	11.694 [5.769;17.644]	**<0.001**
	Uncertainty	160	32.1 [19.5]	78	30.3 [17.3]	82	33.6 [21.5]	3.309 [-3.226;9.478]	0.321
	Effective decision	160	21.2 [18.9]	78	17.5 [14.4]	82	24.8 [21,6]	7.347 [1.499;13.448]	**0.021**

DCS: Decisional Conflict Scale, SD: standard deviation, IG: intervention group, CG: control group. Statistical significance: two-sided independent-samples t-test with bootstrapping. Significant p-values are shown in bold.

At T1, there were significant differences in the mean DCS scores between IG and CG in the subscores support and values clarity. Both mean DCS subscores were significantly higher in the IG.

At T2, mean DCS total scores and the mean subscores informed, support and effective decision were significantly lower in the IG compared to the CG. In the IG, except for the uncertainty subscore (mean 30.3 [17.3]), all scores decreased to values < 25, indicating a mild conflict. In the CG, except for the informed subscore (mean 22.9 [19.8]), all scores were > 25, indicating a moderate conflict.

Multiple regression analysis confirmed the trends observed (Table 6): While the multiple regression model for the DCS total score at T2 showed a positive non-significant association with family planning for the CG (b = 2.199, SE = 5.117, p = 0.668), this association changed to negative in the IG (b = −4.371, SE = 2.813, p = 0.122), meaning that women with open family planning, who participated in the DC program, had less decisional conflict at T2 compared to women with completed family planning.

**Table 6:** Linear regression model*: DCS total score at T2 in the IG and CG.

Independent variables	R²	Beta	SE	t	p-value
*Model 1: IG*	*0.066*				
Family planning (completed vs. open)		**−4.371**	**2.813**	**−1.554**	**0.122**
Children (yes vs. no)		2.547	2.644	0.964	0.337
Family status (single vs. in a relationship)		−3.664	2.450	−1.496	0.137
Employment status (unemployed vs. employed)		−4.918	2.816	−1.747	0.083
Educational level (non-academic vs. academic)		0.368	1.986	0.185	0.853
Age		0.178	0.164	1.086	0.279
*Model 2: CG*	*0.058*				
Family planning (completed vs. open)		**2.199**	**5.117**	**0.430**	**0.668**
Children (yes vs. no)		−3.023	4.210	0.505	0.614
Family status (single vs. in a relationship)		1.172	3.542	0.223	0.824
Employment status (unemployed vs. employed)		4.440	3.700	1.483	0.139
Educational level (non-academic vs. academic)		2.459	3.067	2.070	**0.039**
Age		−0.430	0.240	−0.606	0.545

*Dependent variable = DCS total score at T2. R²: coefficient of determination, Beta: non-standardized beta coefficient, SE: standard error, t: t-statistic. Significant p-values are shown in bold.

With regard to the educational level, the positive significant association between the educational level and the DCS total score that was observed at T1 (Table 3) was still present at T2 in the CG (Table 6). This means that women in the CG with an academic status had a significantly higher decisional conflict than those with a non-academic status. In the IG, the trend was observed as well at T2 but the association was not significant.

### 3.3. Decision status

#### 3.3.1. Decision status of the total group.

As Stock et al. had reported [21], decision status at T1, T2, and T3 comparing IG and CG in the total group was as follows: At T1, 38.6% of the total group stated that they have already decided on one of the three preventive options, with no difference between IG and CG.

At T2, 62.8% of the total group have already decided on a preventive option. There was a significant difference between the IG and the CG (71.4% decided in the IG vs. 54.7% decided in the CG; *p* = 0.001).

At T3, 68.1% of the total group have already decided on a preventive option. There was a significant difference between the IG and the CG (75.1% decided in the IG vs. 61.1% decided in the CG; *p* = 0.005).

#### 3.3.2. Decision status of women with open and completed family planning.

Decision status was compared between women with open family planning and those with completed family planning. At T1, there was no significant difference in decision status between the subgroups (n = 68, 36.2% of women in the open family planning group had made a decision, compared to n = 72, 45% in the completed family planning group; p = 0.094).

IG and CG were compared within both subgroups (complete data is listed in additional file C). At T1, decision status of IG and CG were comparable both within the subgroup of women with open family planning and within the subgroup of women with completed family planning.

Within the subgroup of women with completed family planning, there was no significant difference in the decision status between IG and CG neither at T2 nor at T3.

**Fig 2:**
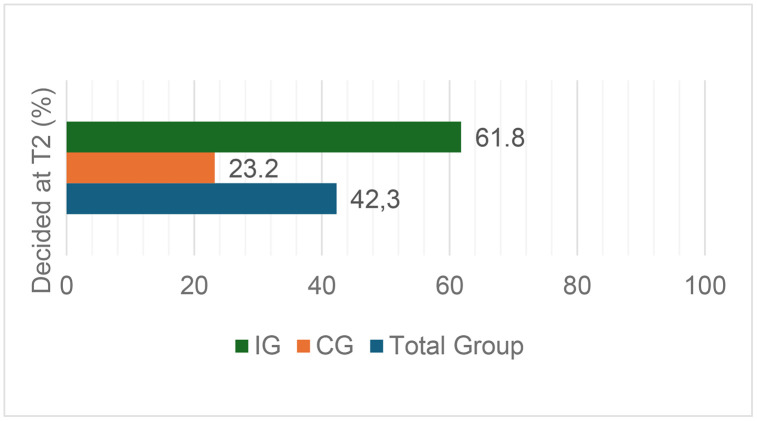
Women with open family planning and undecided at T1. IG=Intervention Group, CG= Control Group, Total Group= IG+CG, Complete data is listed in additional file D.

Within the subgroup of women with open family planning, significantly more women in the IG had decided on a preventive option at T2 and at T3 compared to the CG. To get a closer look on the women who were decided at T2 but had still been undecided at T1, differences in decision status at T2 between IG and CG were analyzed particularly for the subgroup of women with open family planning that were undecided at T1 (complete data is listed in additional file D). Results are illustrated in Fig 2, comparing IG, CG and the total group. In the IG, almost three times as many women (61.8%) were decided at T2 than in the CG (23.2%).

Table 7 shows which preventive option women chose 12 weeks after study inclusion, comparing IG and CG both within the subgroups of women with open family planning and with completed family planning.

**Table 7:** Preventive decision at T2 of women with open and completed family planning.

		OFP				CFP			
		Total	IG	CG	p	Total	IG	CG	p
IBS	Yes, n (%)	86 (45.7)	57 (58.2)	29 (32.2)	**<0.001**	77 (48.1)	40 (51.3)	37 (45.1)	0.436
	No, n (%)	102 (54.3)	41 (41.8)	61 (67.8)		83 (51.9)	38 (48.7)	45 (54.9)	
	Total, n (%)	188 (100)	98 (100)	90 (100)		160 (100)	78 (100)	82 (100)	
RRBM	Yes, n (%)	59 (31.4)	36 (36.7)	23 (25.6)	0.099	37 (23.1)	16 (20.5)	21 (25.6)	0.445
	No, n (%)	129 (68.6)	62 (63.3)	67 (74.4)		123 (76.9)	62 (79.5)	61 (74.4)	
	Total, n (%)	188 (100)	98 (100)	90 (100)		160 (100)	78 (100)	82 (100)	
RRBSO	Yes, n (%)	56 (29.8)	35 (35.7)	21 (23.3)	0.064	90 (56.2)	43 (55.1)	47 (57.3)	0.780
	No, n (%)	132 (70.2)	63 (64.3)	69 (76.7)		70 (43.8)	35 (44.9)	35 (42.7)	
	Total, n (%)	188 (100)	98 (100)	90 (100)		160 (100)	78 (100)	82 (100)	

T2: 12 weeks after study inclusion, IG: intervention group, CG: control group, OFP: open family planning, CFP: completed family planning, p: p-value, IBS: intensified breast surveillance, RRBM: risk-reducing bilateral mastectomy, RRBSO: risk-reducing bilateral salpingo-oophorectomy. Statistical significance: 2-sided Pearson-Chi-Quadrat.

In the subgroup of women with completed family planning, slightly more women in the IG decided for participating in the IBS program and less women opted for RRBM or RRBSO in the IG compared to the CG, but these trends were not significant.

In the subgroup of women with open family planning, significantly more women in the IG decided for participating in the IBS program compared to the CG. At the same time, more women in the IG opted for RRBM or RRBSO compared to the CG, but these trends did not reach statistical significance.

## 4. Discussion

This exploratory sub-analysis of an RCT evaluated a DC program especially developed for unaffected female *BRCA1/2* PV carriers in Germany. It assessed the program’s impact on decisional conflict and decision status of women with open family planning and completed family planning. Our results indicate that the DC program has the potential to considerably reduce decisional conflict and increase decision-making, particularly among women with open family planning. However, given the exploratory and post hoc nature of the subgroup analyses, findings should primarily be interpreted as hypothesis-generating.

In the total group (including both women with open family planning and completed family planning), 12 weeks after study start, DCS total scores and all subscores decreased significantly more in the IG, who participated in the DC program, than in the CG receiving UC. A previous study [26] that evaluated the effects of the DA only, which is part of this DC program and was specially developed for unaffected *BRCA1/2* PV carriers, showed a decrease in the DCS total score and only two subscores, informed and support, in the IG compared to the CG 12 weeks after DA use. Comparing these results to the results of the present study, it can be concluded that the combined implementation of a targeted DC with an evidence-based DA specially developed for unaffected *BRCA1/2* PV carriers can reduce decisional conflict to a broader and stronger extent: Using the DC program including the DA, decisional conflict can be reduced even more, women were more certain, clearer about their values and perceived their decisions more effective. To the best of our knowledge, there is no study focusing on the effect of DC programs on decisional conflict of unaffected *BRCA1/2* PV carriers and the present study’s results indicate a clear impact of the targeted DC program on the decisional conflict of these women.

At the beginning of the present study, women with open family planning experienced higher decisional conflict, felt less informed and were less clear about their personal values regarding benefits and risks/side effects compared to women with completed family planning. While women with completed family planning had a high score in the DCS subscale uncertainty only, women with open family planning had scores that can be categorized as high in the total scale and the subscales values clarity, uncertainty and effective decision at the beginning of the study. These results reflect the situation of this particular subgroup: Women who carry a *BRCA1/2* PV and have not yet completed their family planning struggle not only with preventive decisions regarding their cancer risks, but also with reproductive decisions on whether, when and how to become a mother [34,35]. Fertility-related concerns as well as the need to complete family planning early have a significant psychological impact on these women [36, 37]. On the one hand, preventive surgeries minimize the BC/OC risks, but on the other hand they impair fertility and/or the ability to breastfeed. Dean et al. [36] examined unaffected *BRCA1/2* PV carriers’ decision-making styles about family planning and risk management. The authors identified logical decision-making, which prioritized decreasing the personal risks of BC/OC over having children, and emotional decision-making, which means prioritizing having children by extending the preventive surgery timeline. Benedict et al. [38] evaluated associations among reproductive distress, avoidance, and family-building decision-making of adolescent and young adult female cancer survivors. In their study, higher levels of reproductive distress were related to higher levels of decisional conflict (DCS total score). The authors stated that decision-making support or counseling might be helpful in the decision-making process for these women.

After 12 weeks, women with an open family planning who had participated in the DC program had a significant decrease in the DCS total score and all subscores compared to women with an open family planning receiving UC. In the IG participating in the DC program, all scores decreased to a mild conflict, whereas scores of women of the CG were still moderate or high. This indicates that the DC program can help women with an open family planning to feel better informed and supported, more certain, clearer about their values and to perceive their decision as more effective. In contrast, women with completed family planning who participated in the DC program had a reduced score in the DCS total scale and the DCS subscales informed and support only compared to the CG receiving UC. This is in line with the results for the DA used within the DC program in terms of decision-related outcomes: There was also a decrease in the total score and the subscores informed and support in the IG compared to the CG 12 weeks after DA use [26]. The results of the present analysis indicate that the DC program might help to reduce decision conflict (total score and all subscores) to a considerably stronger extent in the subgroup of women with open family planning compared to the subgroup with completed family planning.

With respect to decision status, there was no difference between women with open family planning and completed family planning at baseline. After 12 weeks, in the subgroup of women with open family planning significantly more women in the IG stated to be decided on a preventive option compared to the CG. This suggests that participating in the DC program can enhance decision-making in initially undecided women with open family planning considerably more than receiving UC. Furthermore, significantly more women in the IG decided to participate in the IBS program compared to the CG. Since the IBS program is an option that has no impact on childbearing and breast feeding, this might indicate that the DC program may be able to help women with open family planning to include reproductive considerations in their preventive decision-making. However, the avoidance of irreversible options might also be a factor that has influenced the decision to participate in the IBS program. In the subgroup of women with completed family planning, there was no significant difference between IG and CG regarding the decision status and regarding the decision for IBS participation. The findings suggest that the DC program may address specific decisional support needs of women with open family planning and may particularly help these women to reach a preventive decision.

According to our results, the DC program seems to be particularly helpful for the subgroup of unaffected *BRCA1/2* PV carriers with open family planning to better cope with this complex and emotionally demanding decision-making situation and manage their decision-making process. Clinicians should take this effect into account during their very first contact with women seeking advice. During genetic counseling of healthy women with *BRCA1/2* PV – which is often the first interaction with these women – family planning should be specifically addressed and discussed in order to identify those women who could particularly benefit from the DC program. This could be particularly helpful if financial and human resources are insufficient to provide participation in a DC program to all unaffected *BRCA1/2* PV carriers.

### 4.1. Strengths and limitations

A clear strength of this study is that the analyses were performed using the data set of a prospective multicenter RCT including a large number of participants. This suggests a high level of validity. Since the original trial was conducted in the context of real-world counseling and care in GC-HBOC centers, good transferability to clinical practice can also be expected. Furthermore, this study addresses a clinically relevant subgroup of women with *BRCA1/2* PVs who face two complex, interrelated and far-reaching decisional issues: deciding on both one’s own preventive strategy and personal family planning.

Several limitations should be considered when interpreting the findings. First, this study represents an exploratory post hoc subgroup analysis and therefore carries an increased risk of chance findings. Second, baseline characteristics differed between women with open family planning and those with completed family planning. Although adjusted regression models were applied, residual confounding cannot be excluded. In addition, missing follow-up data may have been associated with decisional conflict itself, potentially contributing to selective non-response bias.

Furthermore, given the exploratory character of the analyses and the large number of statistical tests performed, the results should primarily be interpreted as hypothesis-generating. No formal adjustment for multiple testing was applied; therefore, the findings require cautious interpretation. Despite these limitations, the study provides initial insights into decisional conflicts experienced by women with *BRCA1/2* PVs and suggests that the DC program may support decision-making, particularly in women with open family planning. Further prospective studies with predefined hypotheses are needed to confirm these findings.

## 5. Conclusions

In conclusion, the present sub-analyses indicate that the DC program for unaffected *BRCA1/2* PV carriers may considerably contribute to reducing decisional conflict and increasing decision-making especially in women with open family planning. Considering the difficult and complex reproductive and preventive decisions that these women face, the DC program can be a valuable and more patient-centered addition to the current care and counseling concept for unaffected women with a *BRCA1/2* PV in the German healthcare system.

## Supporting information

S1 File Supplementary Tables. (DOCX)

## Abbreviations

BC: Breast cancer
CFP: Completed family planning
CG: Control group
DA: Decision aid
DC: Decision coaching
DCS: Decisional conflict scale
GC-HBOC: German Consortium for Hereditary Breast and Ovarian Cancer
IBS: Intensified breast surveillance
IG: Intervention group
OC: Ovarian cancer
ODSF: Ottawa Decision Support Framework
OFP: Open family planning
PV: Pathogenic variant
RCT: Randomized controlled trial
RRBM: Risk-reducing bilateral mastectomy
RRBSO: Risk-reducing bilateral salpingo-oophorectomy
SDM-S: Stage of decision-making scale
UC: Usual care
