# Supplementary material for: Decision coaching for healthy women with BRCA1/2 pathogenic variants and open family planning: Impact on decisional conflict and decision status: Subgroup analyses from a randomized controlled trial
Source: PLoS One. 2026 Aug 17;21(8):e0354884. doi: 10.1371/journal.pone.0354884 (PMC13480602; doi:10.1371/journal.pone.0354884)
Supplement: Supplementary file 1 [file pone.0354884.s001.docx]

**Additional files**

**Additional file A**

| **Tab A** Baseline characteristics of women with open and completed family planning | | | | | |
| --- | --- | --- | --- | --- | --- |
|  | Total  (n=348) | OFP  (n=188, 54.0%) | CFP  (n=160, 46.0%) | Difference OFP-CFP [95% KI] | p-value |
| Age in years, mean [SD] | 35.9 [6.1] | 29.4 [4.1] | 42.4 [8.1] | -12.9  [11.616; 14.379] | **<0.001** |
| Pathogenic variant PV, n (%)* | 343 (100.0) | 186 (100.0) | 157 (100.0) |  |  |
| *BRCA1* PV, n (%) | 167 (48.7) | 97 (52.2) | 70 (44.6) | 7.6  [-0.031;0.175] | 0.153 |
| *BRCA2* PV, n (%) | 176 (51.3) | 89 (47.8) | 87 (55.4) |  |  |
| Family status, n (%) | 348 (100) | 188 (100) | 160 (100) |  |  |
| Single, n (%) | 153 (44.0) | 119 (63.3) | 34 (21.2) | 42.1  [0.329;0.527] | **<0.001** |
| In relationship, n (%) | 195 (56.0) | 69 (36.7) | 126 (78.8) |  |  |
| Children, n (%) | 348 (100) | 188 (100) | 158 (100) |  |  |
| Yes, n (%) | 176 (50.6) | 45 (23.9) | 130 (82.3) | 58.4  [-0.664;0.496] | **<0.001** |
| No, n (%) | 172 (49.4) | 143 (76.1) | 28 (17.7) |  |  |
| Employment status, n (%) | 348 (100) | 188 (100) | 160 (100) |  |  |
| Employed, n (%) | 279 (80.2) | 144 (76.6) | 135 (84.4) | 7.8  [-0.002;0.156] | 0.062 |
| Unemployed, n (%) | 69 (19.8) | 44 (23.4) | 25 (15.6) |  |  |
| Education level, n (%) | 346 (100) | 188 (100) | 158 (100) |  |  |
| Academic, n (%) | 160 (46.2) | 104 (55.3) | 56 (35.4) | 19.9  [-0.297;-0.092] | **<0.001** |
| Non-academic, n (%) | 186 (53.8) | 84 (44.7) | 102 (64.6) |  |  |
| *SD: standard deviation, PV: pathogenic variant, OFP: open family planning, CFP: completed family planning, missing values result from missing information in the questionnaires. *PV was not documented for 7 women.* | | | | | |

**Additional file B**

| **Tab B** Decisional Conflict Scale at baseline (T1), 12 weeks (T2) and 6 months (T3) after study inclusion | | | | | | | |
| --- | --- | --- | --- | --- | --- | --- | --- |
|  |  | IG | | CG | | Difference CG-IG [95% KI] | p-value |
|  |  | n | mean [SD] | n | mean [SD] |  |  |
| T1 | Total score | 196 | 39.2 [18.8] | 191 | 37.1 [20.9] | -2.1  [-5.888;2.808] | 0.408 |
|  | Informed | 196 | 33.6 [20.3] | 191 | 32.5 [20.3] | -1.1  [-5.392;3.964] | 0.705 |
|  | Values clarity | 196 | 40.5 [23.2] | 191 | 39.9 [25.8] | -0.6  [-6.093;4.340] | 0.662 |
|  | Support | 196 | 38.2 [19.4] | 191 | 33.6 [21.4] | -4.6  [-8.771;0.225] | 0.568 |
|  | Uncertainty | 196 | 47.1 [24.2] | 191 | 45.3 [26.1] | -1.9  [6.094;4.579] | 0.742 |
|  | Effective decision | 196 | 37.4 [20.9] | 191 | 35.8 [22.4] | -1.6  [-5.565;3,781] | 0.646 |
| T2 | Total score | 174 | 16.7 [12.0] | 183 | 30.1 [18.8] | 13.4  [10.165;16.842] | **<0.001** |
|  | Informed | 174 | 10.3 [12.1] | 183 | 25.9 [19.6] | 15.6  [12.458;19.322] | **<0.001** |
|  | Values clarity | 173 | 16.2 [15.7] | 183 | 30.8 [22.9] | 14.6  [10.318;18.841] | **<0.001** |
|  | Support | 172 | 13.8 [14.8] | 183 | 28.5 [20.6] | 14.7  [10.861;18.443] | **<0.001** |
|  | Uncertainty | 171 | 26.9 [17.6] | 183 | 37.4 [23.3] | 10,5  [6.270;15.070] | **<0.001** |
|  | Effective decision | 172 | 16.6 [13.9] | 183 | 28.3 [21.1] | 11.7  [8.058;15.770] | **<0.001** |
| T3 | Total score | 173 | 16.0 [11.9] | 175 | 28.0 [18.3] | 12  [8.635;15.299] | **<0.001** |
|  | Informed | 173 | 10.2 [12.6] | 175 | 25.8 [20.2] | 15.6  [11.663;18.741] | **<0.001** |
|  | Values clarity | 173 | 14.2 [16.2] | 175 | 26.2 [21.3] | 12  [8.063;16.151] | **<0.001** |
|  | Support | 173 | 13.7 [14.4] | 175 | 26.6 [19.5] | 12.9  [9.275;16.704] | **<0.001** |
|  | Uncertainty | 173 | 25.5 [16.9] | 175 | 34.0 [22.2] | 8.5  [4.145;12.817] | **0.001** |
|  | Effective decision | 173 | 16.3 [13.9] | 175 | 27.6 [20.3] | 11.3  [7.219;14.817] | **<0.001** |
| *T1: baseline, T2: 12 weeks after study inclusion, T3: 6 months after study inclusion, DCS: Decisional Conflict Scale, SD: standard deviation, IG: intervention group, CG: control group. Statistical significance: two-sided independent-samples t-test with bootstrapping. Significant p-values are shown in bold.* | | | | | | | |

**Additional file C**

| **Tab C** Decision status of women with open and completed family planning | | | | | | | | | |
| --- | --- | --- | --- | --- | --- | --- | --- | --- | --- |
|  |  | OFP | | | | CFP | | | |
|  |  | Total | IG | CG | p-value | Total | IG | CG | p-value |
| T1 | Decided, n(%) | 68 (36.2) | 35 (35.7) | 33 (36.7) | 0.913 | 72 (45.0) | 32 (41.0) | 40 (48.8) | 0.446 |
|  | Undecided, n(%) | 120 (63.8) | 63 (64.3) | 57 (63.3) |  | 88 (55.0) | 46 (59.0) | 42 (51.2) |  |
|  | Total, n(%) | 188 (100.0) | 98 (100.0) | 90 (100.0) |  | 160 (100.0) | 78 (100.0) | 82 (100.0) |  |
| T2 | Decided, n(%) | 99 (57.2) | 60 (69.8) | 39 (44.8) | **0.002** | 101 (67.8) | 48 (68.6) | 53 (67.1) | 0.896 |
|  | Undecided, n(%) | 74 (42.8) | 26 (30.2) | 48 (55.2) |  | 48 (32.2) | 22 (31.4) | 26 (32.9) |  |
|  | Total, n(%) | 173 (100.0) | 86 (100.0) | 87 (100.0) |  | 149 (100.0) | 70 (100.0) | 79 (100.0) |  |
| T3 | Decided, n(%) | 100 (59.5) | 60 (71.4) | 40 (47.6) | **0.004** | 108 (75.0) | 55 (78.6) | 53 (71.6) | 0.451 |
|  | Undecided, n(%) | 68 (40.5) | 24 (28.6) | 44 (52.4) |  | 36 (25.0) | 15 (21.4) | 21 (28.4) |  |
|  | Total, n(%) | 168 (100.0) | 84 (100.0) | 84 (100.0) |  | 144 (100.0) | 70 (100.0) | 74 (100.0) |  |
| *T1: Baseline, T2: 12 weeks after study inclusion, T3: 6 months after study inclusion, IG: intervention group, CG: control group, OFP: open family planning, CFP: completed family planning. Statistical significance: 2-sided Mann-Whitney-U-Test. P-values were corrected for multiple testing by using the Benjamini-Hochberg correction.* | | | | | | | | | |

**Additional file D**

| **Tab D** Decision status of women that were undecided at T1 | | | | |
| --- | --- | --- | --- | --- |
|  | Women with open family planning and undecided at T1 | | | |
|  | Total group | IG | CG | p-value |
| Decided at T2, n(%) | 47 (42.3) | 34 (61.8) | 13 (23.2) | **<0.001** |
| Undecided at T2, n(%) | 64 (57.7) | 21 (38.2) | 43 (76.8) |  |
| Total, n(%) | 111* (100.0) | 55 (100.0) | 56 (100.0) |  |
| *T1: Baseline, T2: 12 weeks after study inclusion, IG: intervention group, CG: control group. Statistical significance: 2-sided Pearson-Chi-Quadrat. P-values were corrected for multiple testing by using the Benjamini-Hochberg correction. *Of those undecided at T1 (n=120) only 111 completed this question at T2.* | | | | |
